# Neurodevelopmental Outcome in Extremely Low Birth Weight Infants at 2–3 Years of Age

**DOI:** 10.3390/medicina56120649

**Published:** 2020-11-26

**Authors:** Maria Kyriakidou, Ilias Chatziioannidis, Georgios Mitsiakos, Sofia Lampropoulou, Abraham Pouliakis

**Affiliations:** 1 Occupational Therapy Department, University of Western Macedonia, 50100 Kozani, Greece; 2 2nd Neonatology Department, Aristotle University, 56403 Thessaloniki, Greece; eanosmg@gmail.com; 3 Physiotherapy Department, School of Health Rehabilitation Sciences, University of Patras, 25100 Aigio, Greece; szlampropoulou@gmail.com; 4 2nd Department of Pathology, National and Kapodistrian University of Athens, 12462 Athens, Greece; apou1967@gmail.com

**Keywords:** ELBW, HINE, Bayley III, neurodevelopmental outcome, infants

## Abstract

*Background and objectives:* The aims of this study were to examine the relationship between neurological outcomes at 3- and 6-months corrected age with the neurodevelopmental outcome at 3 years of age; to identify the perinatal/neonatal risk factors for poor neurodevelopmental outcomes at 3 years of age. *Materials and methods:* In our single-centre longitudinal cohort study, of the 73 consecutive infants admitted to our Neonatal Intensive Care Unit (NICU), 49 infants (80%) received both Hammersmith Infant Neurological Examination (HINE) at 3- and 6-months corrected age and Bayley–III neurodevelopmental assessment at 2–3 years chronological age. At 3 months follow up, 8.2% had suboptimal scores (below 10th percentile) on the HINE. At 6 months follow up, 4.1% had suboptimal scores (below 10th percentile) on the HINE. The means(±SD) for Bayley-III cognitive, language, and motor subscales were (96.3 ± 9.8), (99.9 ± 11.9), (93.2 ± 9.9). *Results:* At 3 months corrected age, higher total HINE scores and subscores for function of cranial nerves, posture, tone, were associated with better cognitive scores while poorer scores for function of cranial nerves, posture, movements, tone, and total HINE score were associated with lower motor scores. Infants with a HINE subscore of function of cranial nerves in the suboptimal range have three times higher odds of having a motor delay. Infants with a HINE subscore of function of cranial nerves in the suboptimal range have more than two times higher odds of having a language delay. At 6 months corrected age, poorer scores for function of cranial nerves, movements, tone, reflexes, and total HINE score were associated with worse Bayley-III motor scores whilst infants who have a total HINE score and a subscore of reflexes in the suboptimal range have four and seven times, respectively, higher odds of having a motor delay. *Conclusions:* Early identification of infants at risk for adverse long-term outcomes is essential in introducing early intervention therapies for optimizing neurodevelopmental outcomes.

## 1. Introduction

Over the past two decades, there has been extensive literature on babies with extremely low birth weight (ELBW < 1000 g) and/or extremely preterm (EP < 28 weeks), documenting improved survival mainly due to regionalization of perinatal care, improved technology, and better understanding of their pathophysiology and specific needs [1]. Nevertheless, the incidence of ELBW births remains a major public health problem with considerable medical and financial impact due to the concurrent increase in long-term comorbidities. Rates of major neurodevelopmental impairment varied between 15–40% [2,3,4] among ELBW survivors and include cerebral palsy, mental retardation, visual-auditory deficits; and have remained relatively unchanged [5,6]. In advance, the risk for long-term minor motor, cognitive, and behavioral deficits remains high [7,8].

Thus, comprehensive examination of ELBW/EP infants should include both neurological and neurodevelopmental assessments for fine-gross motor, cognitive, and language skills but also for social-emotional and adaptive behavior functioning, in order to provide the opportunity for enrollment to follow-up program, individualized family training, identification of infants eligible to early intervention services, and yield improved long-term outcomes [9,10].

Hammersmith Infant Neurological Examination (HINE) is a well-studied neurological exam in high-risk infants and can been used both for clinical and research needs; its sequential use also allows the identification of early signs of cerebral palsy and other neuromotor disorders [11].

Among the most widespread tools for neurodevelopmental assessment, the Bayley Scales of Infant and Toddler Development III comprises of three distinct composite scores (Motor, Cognitive, and Language) allows a more accurate assessment of specific developmental domains, and contributes to an in-depth approach to premature infants’ follow-up [12]. However, it has been consistently found that Bayley-III composite scores are up to 10 points higher than those of Bayley-II [13,14]. Hence, in clinical settings, concerns have arisen that the Bayley-III may underestimate developmental impairment [15], reducing the number of children referred to early intervention services.

Although, HINE score and individual items are predictive of motor outcomes, it is unclear whether those scores and items are associated with specific deficits in cognitive, fine-gross motor, or expressive-receptive language domains of neurodevelopment.

Therefore, the aims of this prospective study in a cohort of preterm ELBW infants were firstly, to examine the relationship between neurological assessments at 3 and 6 months corrected age with neurodevelopmental outcome at 3 years of age; secondly, to identify the perinatal/neonatal risk factors for poor neurodevelopmental outcomes at 3 years of age.

## 2. Materials and Methods

### 2.1. Participants

We implemented a single-center longitudinal cohort study. The study design was approved by the Hospital’s Ethics Committee (approval number 46134; approval date 11/11/2015) and written informed consent provided by parents for their infant to participate in the study.

Inclusion criteria included: birth weight ≤1000 g (ELBW) and/or gestational age ≤ 29 + 6 weeks. Infants with genetic abnormalities and/or neurosensory disabilities (blindness, deafness) were excluded from the study.

**Figure 1:**
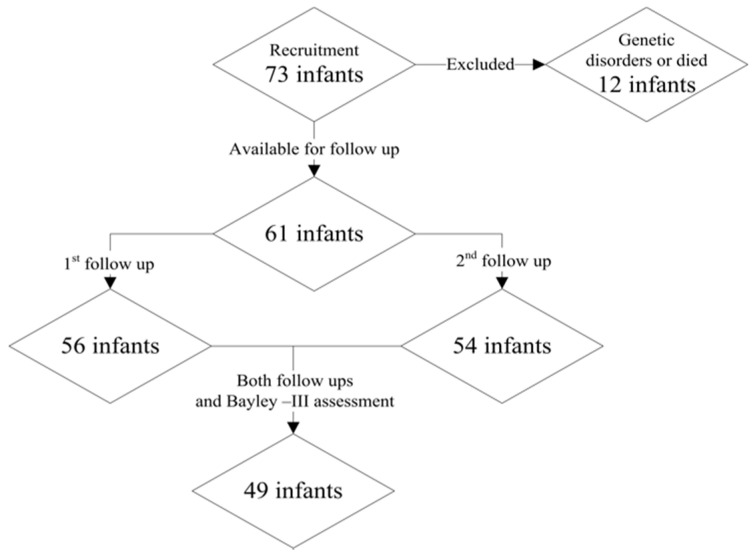
Flow diagram of the study participants.

All the 73 ELBW infants consecutively admitted to our NICU from January 2014 to December 2014, 62 (84%) were discharged home alive. At baseline, 39 infants were males (53.4%) and 34 females (46.5%). Of all 73 infants recruited, 61 infants entered the study, 1 was excluded due to genetic abnormalities and 11 died, 56 (91.8%) returned for the 3 months corrected age follow-up visit, 54 (88.5%) for the 6 months corrected age follow-up visit. In total, 49 infants (80%) received both HINE neurological assessments (at 3- and 6-months corrected age) and Bayley–III neurodevelopmental assessment at 2–3 years chronological age (Figure 1).

Study data were collected prospectively and included: infant’s sex, gestational age (weeks), and weight (grams) at birth, head circumference. Other neonatal variables included: Apgar scores at 5 min, singleton, twin or multiple pregnancy, mode of delivery, being small for gestational age (SGA), and length of hospital stay. Clinical variables such as respiratory distress syndrome, duration of invasive mechanical ventilation, days on oxygen, postnatal surfactant treatment, sepsis, patent ductus arteriosus (PDA), bronchopulmonary dysplasia, necrotizing enterocolitis, retinopathy of prematurity (ROP), and complications during the hospital stay such as intraventricular hemorrhage the highest grade documented (IVH), periventricular leukomalacia (PVL) were recorded. Maternal characteristics included chorioamnionitis, antenatal corticosteroid therapy, preeclampsia/eclampsia, chorioamnionitis, gestational diabetes, and intrauterine growth retardation (IUGR).

Gestational age was determined from the date of the last menstrual period and was confirmed by early ultrasound examination. BW was measured with an electronic scale. Infants were classified as small for gestational age according to the Fenton Growth Chart [16]. Neonatal sepsis was considered in the presence of a positive blood and/ cerebrospinal fluid culture. Necrotizing enterocolitis (NEC) NEC was defined as clinical gastrointestinal disturbances associated with pneumatosis intestinalis on abdominal radiography [17]. BPD was defined by the use of oxygen at 36 weeks of postmenstrual age. IVH and PVL were detected by brain ultrasound scans. All infants underwent early cranial ultrasound scans (<72 h of life) and underwent a further cranial ultrasound at discharge. Cranial ultrasound scans were routinely performed during the neonatal period by a neonatologist and were classified as ‘normal’ or ‘abnormal’ if flares, ventricular dilatation, porencephalic cysts, or atrophy were present, alone, or in combination. Corrected age was calculated by subtracting from the chronological age the time left before term.

Infants were scheduled to be examined at 3, 6 months corrected age and 36 months chronological age. Hammersmith Infant Neurological Examination (HINE) was performed at 3- and 6-months appointments, after a standardized physical examination. An experienced neonatologist and a trained pediatric physiotherapist performed neurological and neurodevelopmental assessments.

HINE assessment consists of 26 items exploring the areas of cranial nerve function, posture, quality, and quantity of movements, tone, and reflexes/reactions. Each item is scored individually; the examiner marks the most relevant of the four columns scoring 0,1,2,3, and scores can be added to achieve a global optimality score, range: 0–78. The assessment has been standardized in very preterm infants between 6- and 15-months corrected age [18], and also the obtained reference cut-off values for the optimality scores have been validated in a population of very preterm infants from 3 to 12 months post-term age [19]. In typically developed infants, optimality scores are based on the frequency distribution of neurological findings and an item is considered optimal when it is marked in 90% of infants [20]. In each follow-up appointment, motor milestones were marked in the second part of HINE.

At 36 months chronological age a pediatric physiotherapist, unaware of the child’s perinatal history, applied neurodevelopmental assessment using Bayley-III scales to all infants. The third edition of the Bayley Scales of Infant and Toddler Development is a valid, reliable, and standardized assessment tool of developmental delay [12]. It encompasses three scales: Cognitive (composite index score: range 55–145), which emphasizes on the mental development through methods that minimize language involving sensorimotor development, exploration and manipulation, object relatedness, concept formation, memory and simple problem solving; language (composite index score: range 45–155), which has expressive (babbling, gesturing and utterances) and receptive (verbal comprehension, vocabulary) subscales; and motor (composite index score: range 45–155), which assesses gross motor (head control, sitting, stepping, standing, walking, climbing, and running) and fine motor (prehension, perceptual-motor integration, motor planning and speed, reaching, functional hand skills, and response to tactile information) subscales separately.

Bayley III, in all three composite index scores, has index mean scores of 100(SD ± 15). An index composite score of <70 (>2 SD below the mean) is defined to indicate severe impairment, while an index composite score of 71–85 (>1 SD below the mean) is defined to indicate mild impairment. According to Bayley III, index composite scores ≥86 indicate normal development [12].

### 2.2. Statistical Analysis

The Statistical Package for the Social Sciences (SPSS Inc. Released 2008. SPSS Statistics for Windows, Version 17.0. SPSS Inc.: Chicago, IL, USA) was used for data analysis. The relation between categorical variables was investigated using χ^2^ or Fisher exact tests. To determine the relation between continuous variables, the Mann-Whitney or the t test was used depending on the distribution. Fisher exact test was used to examine the association between HINE suboptimal subscores and total scores at 3- and 6-months corrected age and developmental delay on Bayley III at 3 years chronological age. Linear regression analysis was used to examine the relation between perinatal/neonatal characteristics of ELBW infants and Bayley III scores in motor, cognitive, and language domains at 2-3 years chronological age. Results are reported as relative risks (RR) and 95% confidence intervals. *p* < 0.05 was considered statistically significant.

## 3. Results

### 3.1. Characteristics of Study Participants

Table 1 depicts perinatal and neonatal characteristics.

The mean age at testing was at a corrected age of mean(±SD) 3.1(±0.85) months and 6.8(±1.44) in the first and second follow up visit with HINE examination, respectively, while the mean age at Bayley–III testing was at 30.9(±7.29) months chronological age.

As shown in Table 1, infants who completed the study were not significantly different to those who were lost to follow-up with the exception of a much higher percentage of multiple pregnancies and RDS in the first group.

Four infants had cerebral palsy, and according to Gross Motor Function Classification System (GMFCS) one was diagnosed with hemiplegia/level I, 2 had diplegia/level I, and one had quadriplegia/level III [21].

Neurodevelopmental outcomes for cognitive, language, and motor domains are reported in Table 2. At 3 years follow up, the percentage of infants that scored more than 1 SD below the mean was 20.4% in cognitive outcome, 10.4% in receptive/expressive language subscale, and 20.4% in fine/gross motor domain (Table 3).

Overall at 3 months, follow up 8.2% had suboptimal scores (below 10th percentile) on the HINE with 4.1% for Function of cranial nerves, 2% for Posture, 4.1% for Movements, 10.2% for Tone, and 2% for Reflexes and reactions.

At 6 months follow up, 4.1% had suboptimal scores (below 10th percentile) on the HINE with 12.2% suboptimal for Function of cranial nerves, 6.1% for Posture, 4.1% for Movements, 8.2% for Tone, and 6.1% for Reflexes and reactions. The median (range) for the HINE at 3- and 6-months corrected age is reported on Table 4.

### 3.2. Association between Hammersmith Infant Neurological Examination Testing and Bayley-III Scales Scores

#### 3.2.1. Cognitive Performance

At 3 months corrected age, better (higher) scores for function of cranial nerves (coefficient: 0.25; 95% CI −0.2–3.50, *p* = 0.082), posture (coefficient: 0.27; 95% CI −0.06–2.78, *p* = 0.059), tone (coefficient: 0.37; 95% CI −0.32–2.20, *p* = 0.01), and total HINE score (coefficient: 0.30; 95% CI −0.04–0.82, *p* = 0.03), were associated with improved cognitive composite score, while poor total HINE score and sub-scores were associated with lower cognitive performance (Table 5).

At 6 months corrected age, no evidence of relationships between HINE total score and sub-scores with cognitive outcome emerged (Table 6).

#### 3.2.2. Language Performance

Both at 3- and 6-months corrected age, no evidence of relationships between HINE total score and sub-scores with receptive/expressive language performance emerged (Table 5 and Table 6).

**Figure 2:**
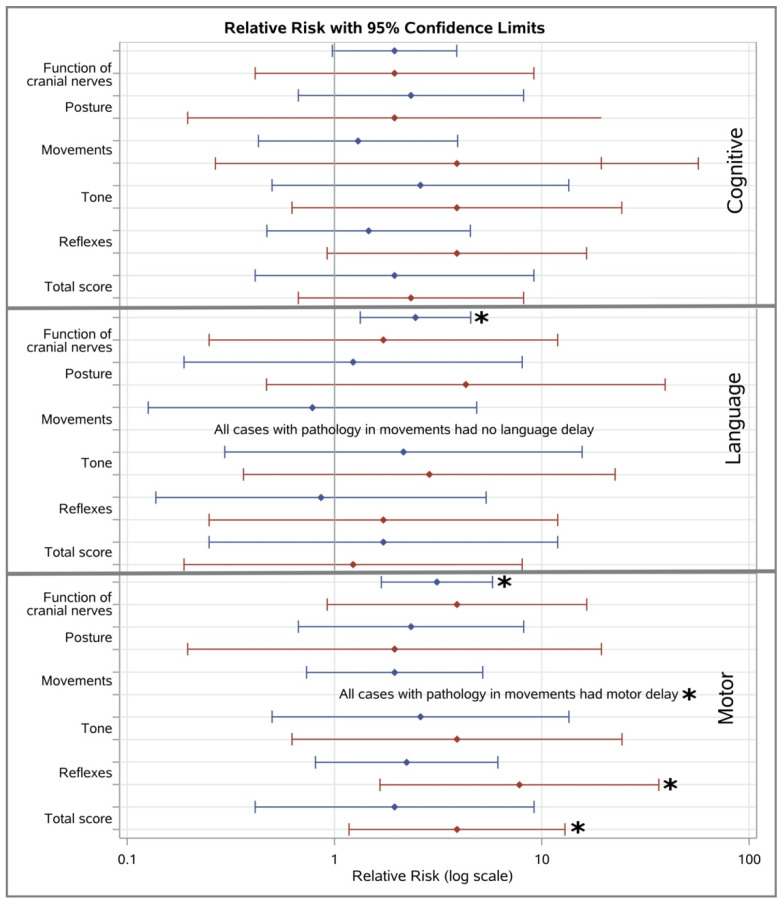
Association between suboptimal total HINE scores/sub-scores at 3- (blue line), 6-months (red line) with cognitive, language and motor delay; * *p* < 0.05.

At 3 months corrected age, suboptimal scores for function of cranial nerves (RR 2.46; 95% CI 1.33–4.54, *p* = 0.03) were associated with increased risk of language delay (Figure 2).

#### 3.2.3. Motor Performance

At 3 months corrected age, poorer scores for function of cranial nerves (coefficient: 0.45; 95% CI 1.2–4.30, *p* = 0.001), posture (coefficient: 0.26; 95% CI −0.10–2.80, *p* = 0.07), movements (coefficient: 0.37; 95% CI 1.00–6.50, *p* = 0.009), tone (coefficient: 0.32; 95% CI −0.13–2.00, *p* = 0.02), and total HINE score (coefficient: 0.39; 95% CI −0.16–0.94, *p* = 0.006) were associated with lower composite motor scores at 3 years of age (Table 5).

At 6 months corrected age, poorer scores for function of cranial nerves (coefficient: 0.27; 95% CI −0.15–5.15, *p* = 0.06), movements (coefficient: 0.36; 95% CI −1.58–11.70, *p* = 0.01), tone (coefficient: 0.27; 95% CI −0.05–1.84, *p* = 0.06), reflexes (coefficient: 0.25; 95% CI −0.20–2.80, *p* = 0.09), and total HINE score (coefficient: 0.20; 95% CI 0.05–0.90, *p* = 0.029) were associated with lower (worse) motor performance at 3 years of age (Table 6).

At 3 months corrected age, suboptimal scores for the HINE subscale of function of cranial nerves (RR 3.12; 95% CI 1.68–5.79, *p* = 0.001) were associated with increased odds of motor delay on Bayley-III at 3 years of age (Figure 2).

At 6 months corrected age, suboptimal scores for the HINE subscale of reflexes (RR 7.80; 95% CI 1.66–36.70, *p* = 0.002), and HINE total score (RR 3.90; 95% CI 1.18–12.94, *p* = 0.023) were associated with increased risk of motor delay on Bayley-III (Figure 2).

### 3.3. Factors Associated with Bayley-III Scales

Regression analysis indicated that significant independent factors at *p* < 0.05 that potentially influenced the motor composite score in Bayley III test were SGA (*p* = 0.01), prenatal steroids (*p* = 0.002), PDA (*p* = 0.013), and necrotizing enterocolitis (*p* = 0.028). Additionally, necrotizing enterocolitis was found to influence both cognitive and language composite scores (*p* = 0.039 and *p* = 0.004, respectively).

## 4. Discussion

We studied the association of the HINE neurological examination at 3- and 6-months corrected age with Bayley-III in a cohort of EP/ELBW infants at two to three years of chronological age. At 3 months corrected age, higher (better) total HINE scores and higher HINE subscores for function of cranial nerves, posture, tone, were associated with better cognitive performance, while poorer scores for function of cranial nerves, posture, movements, tone, and total HINE score were associated with lower (worse) motor scores. Infants who have a HINE subscore of function of cranial nerves in the suboptimal range have three times higher odds of having a motor delay. The relationship between total HINE score/subscores and language scores is less clear but infants who have a HINE subscore of function of cranial nerves in the suboptimal range have more than two times higher odds of having a language delay.

At 6 months corrected age, poorer scores for function of cranial nerves, movements, tone, reflexes, and total HINE score were associated with lower (worse) Bayley III motor scores whilst infants who have a total HINE score and a subscore of reflexes in the suboptimal range have four and more than seven times, respectively, higher odds of having a motor delay.

According to the scientific literature, HINE is the most frequently used method internationally [22]; it is the quickest neurological examination to perform, easy to administer and score without any prior formal certification process, although training is recommended with an experienced user and online videos available [23,24]. In advance, the optimality scores have been validated in a population of very preterm infants from 3 to 12 months post-term age [19].

Our study has demonstrated that both total score of the HINE and subscale scores alone were strongly related to motor outcomes in Bayley III test and this is consistent with previous findings using HINE, which has shown to be reliable in predicting motor outcome at 2 years of age in very preterm infants assessed between 9 and 18 months of age [18]. In our study, this strong relationship has been demonstrated as early as 3 months corrected age.

We obtained reference cut-off values for the optimality scores at 3- and 6-months corrected age and found that ELBW infants with suboptimal HINE scores/sub-scores, as early as 3 months corrected age have higher odds for delay in the language domain. Accordingly, in a cohort of very preterm infants by Romeo et al. (2009), a high correlation, as early as 3 months onwards, between the HINE and the receptive and expressive language development at 24 months was reported [19].

It has been reported that auditory processing abilities are well developed in infancy, and thus, infants with such deficits are at a higher risk for developing a language disorder [25]. In the subsection of function of cranial nerves in HINE testing, auditory response to a rattle is assessed, and probably a suboptimal score in this subsection related to the item of auditory response is associated with the increased odds for language delay at 2–3 years of age found in our study.

We found that poorer HINE subscores for function of cranial nerves, posture, tone, and total HINE scores were associated with worse cognitive scores. Posture subscale of the HINE tool assesses head, trunk arms, hands, legs, and feet posture and asymmetries in those parts of the body. According to de Groot, in early infancy, the development of adequate motor behavior and sensorimotor interaction could be disrupted by inadequate postural behavior, resulting in a faulty perception–action cycle, thus influencing later cognitive development [26]. Furthermore, if postural control is problematic, fine motor function and especially hand function will be delayed or atypically developed, contributing directly to a faulty praxis, thus affecting infants’ later cognitive development and writing skills [26]. In advance, it has been observed that preterm infants with high active muscle power on the trunk had poorer Bayley scores at 18 months of age [27].

Recognizing early developmental delays is crucial for providing early intervention services because the brain is considered to be especially plastic in the phase occurring after the completion of neuronal migration. The processes of dendritic outgrowth and synapse formation are highly active during this phase [28]. The major advantage of intervening early in life is that early sensorimotor information and experiences may have an effect on the architecture of the brain but also directly affect the way the brain is “wired” [29]. In our department, the Newborn Individualized Developmental Care and Assessment (NIDCAP) principals is applied to all ELBW infants. NIDCAP integrates the infant in the natural intrauterine physiological flexion, eludes pain, stress, and over-stimulation, while it supports self-regulation [30]. After discharge, infants below 32 weeks GA follows systematically physiotherapy NDT/Bobath program once to twice per week until they at least achieve independent walking while occupational/speech therapy is introduced when fine motor delay or atypical social emotional and adaptive behavior or feeding problems/language delay are recognized. NDT/Bobath is a clinical practice model, in which the therapist by using the International Classification of Functioning, Disability, and Health (ICF), with a problem-solving approach and through individualized therapeutic handling activates optimal sensorimotor processing, task performance, and skill acquisition [31]. This policy may account for better neurologic and neurodevelopmental scores with increasing age in all groups.

The strengths of our study include the use of a longitudinal prospective cohort, along with very good follow-up rates.

Our study did not involve a population-based cohort, which may limit the generalizability of findings to other populations. It did reflect care at a regional neonatal intensive care unit. Another limitation of the current study is its monocentric nature. A multi-center study could enable the recruitment of higher number of infants and subsequently stronger associations. It would also be beneficial in a longer-term follow up, as the predictive validity of Bayley-III is moderate for school-age outcomes [32,33].

## 5. Conclusions

The current study has considerable implications both in clinical and research settings. HINE at 3- and 6-months corrected age is associated with cognitive, language, and motor performance at 2–3 years in ELBW infants. HINE assessment is a simple, no-cost tool detecting delayed high-risk infants in motor, cognitive, and language domains. Early identification of ELBW infants at risk for adverse long-term outcomes is crucial for intervening early in life with physiotherapy/occupational therapy programs, optimizing neurodevelopmental outcomes and function. Furthermore, a systematic review of the literature targeted on the relationship between ELBW infants and alterations /deficits in their neuropsychomotor development would be recommended.

## Figures and Tables

**Table 1:** Maternal morbidities, and neonatal characteristics in the study group.

Characteristics of Study Participants	Examined (*n* = 49)	Not-Examined (*n* = 12)	*p*-Value
Gestational age (weeks) ^a^	27.7 (1.85)	27.9 (2.4)	ns
Birthweight (g) ^b^	870 (530–1000)	870 (590–1000)	ns
Head Circumference (cm) ^b^	25 (21–28)	25 (20–26.5)	ns
Gender (male), n(%) ^c^	26 (53.1)	5 (41.7)	ns
Multiparity (n) ^c^	15 (30.6)	1 (8.3)	ns
Multiple gestation, n (%) ^c^	19 (38.8)	1 (8.3)	0.083
Mode of delivery (caesarean section), (n%) ^c^	45 (91.8)	10 (83.3)	ns
Chorioamnionitis, n (%) ^c^	3 (6.1)	1 (8.3)	ns
Preeclampsia/Eclampsia, n(%) ^c^	6 (12.2)	1 (8.3)	ns
Gestational diabetes, n(%) ^c^	3 (6.1)	1 (8.3)	ns
APGAR scores 5min ^b^	8 (1–9)	8 (3–9)	ns
Prenatal corticosteroids, n(%) ^c^	40 (81.6)	8 (66.7)	ns
Small for gestational age, n(%) ^c^	14 (28.6)	5 (41.7)	ns
Intrauterine Growth Retardation, n (%) ^c^	13 (26.5)	4 (33.3)	ns
Respiratory distress syndrome, n(%) ^c^	43 (87.8)	8 (66.7)	0.096
Surfactant administration n(%) ^c^	35 (71.4)	7 (58.3)	ns
Patent ductus arteriosus, n(%) ^c^	10 (20.4)	4 (33.3)	ns
Nosocomial sepsis, n(%) ^c^	37 (75.5)	8 (66.7)	ns
Ventilation days (n) ^b^	5 (0–44)	9.5 (0–67)	ns
O_2_ days (n) ^a^	37.8 (26.6)	33.9 (30.2)	ns
Bronchopulmonary Dysplasia	28 (57.1)	6 (50)	ns
Necrotizing Enterocolitis	3 (6.1)	2 (16.7)	ns
Intraventricular Hemorrhage ΙΙI-IV	6 (12.5)	1 (8.3)	ns
Periventricular Leucomalacia	1 (2)	0	ns
Cerebral Palsy	4 (8.2)	0	ns
Hospital stay (days) ^b^	67 (31–154)	74.5 (43–120)	ns

a. The *t*-test was used for probability value; data presented as mean (±SD); b. Mann—Whitney U test was used; data presented as median (range); c. Fisher exact or χ^2^ exact *t*-test accordingly; data presented as n (%). ns is not significant.

**Table 2:** Neurodevelopmental outcomes for cognitive, language, and motor subscales.

Composite Scores	n	Mean	Range	SD
Cognitive	49	96.3	75–115	9.8
Language	48	99.9	74–135	11.9
Motor	49	93.2	70–112	9.9

**Table 3:** Distribution of composite scores on the Bayley III subscales

Bayley III	Composite Scores	Cognitive Scores, n(%)	Language Scores, n(%)	Motor Scores, n(%)
Above average (+1SD)	>116	0	3 (6.3)	0
Average	86–115	39 (79.6)	40 (83.3)	39 (79.6)
Low average (−1SD)	71–85	10 (20.4)	5 (10.4)	8 (16.3)
Extremely low (−2SD)	56–70	0	0	2 (4.1)

**Table 4:** Median, range of global and subsection scores.

HINE Sections	Median ^1^ (Range)	SD	10th Percentile	Median ^2^ (Range)	SD	10th Percentile
Function of cranial nerves	13 (8–15)	1.66	11	15 (12–15)	1.06	12
Posture	14 (8–16) *	1.94	9	14 (10–18)	1.85	13
Movements	6 (2–6)	0.98	3	6 (4–6)	0.54	4
Tone	16 (7–24) *	2.82	13	18 (10–24)	2.99	14
Reflexes	7 (3–12) **	1.69	4	8 (5–13)	1.89	5
**Total score**	56 (28–68) **	6.98	48	62 (43–76)	6.5	54

^1^ Mean time of assessment 3.3 months (mo); ^2^ Mean time of assessment 7 mo; ** *p* < 0.05 and * *p* < 0.1 between assessments.

**Table 5:** Association between HINE total score at three months and Bayley III performance.

HINE Sections	Cognitive			Language			Motor		
	N	Coeff	CI	N	Coeff	CI	N	Coeff	CI
Function of cranial nerves	49	**0.25**	**−** **0.2,** 3.5 *	48	0.22	−0.45, 3.7	49	**0.45**	**1.1,** 4.3 **
Posture	49	**0.27**	**−0.06**, 2.78 *	48	0.13	−1, 2.5	49	**0.26**	−0.1, 2.8 *
Movements	49	0.08	−2.1, 3.7	48	0.42	−3, 4.1	49	**0.37**	1, 6.5 **
Tone	49	**0.37**	0.32, 2.2 **	48	0.15	−0.56, 1.87	49	**0.32**	−0.13, 2 **
Reflexes	49	0.06	−1.3, 2	48	1	−2.9, 1.2	49	0.1	−1.1, 2.3
Total score	49	**0.3**	−0.04, 0.82 **	48	0.13	−0.28, 0.7	49	**0.39**	−0.16, 0.94 **

* *p* < 0.1, ** *p* < 0.05. Statistical significance is presented in bold.

**Table 6:** Association between HINE total score at six months and Bayley III performance.

HINE Sections	Cognitive			Language			Motor		
	N	Coeff	CI	N	Coeff	CI	N	Coeff	CI
Function of cranial nerves	49	0.06	−2.1, 3.2	48	1	−2.1, 4.4	49	**0.27**	−0.15,5.15 *
Posture	49	0.05	−1.3,1.8	48	−0.53	−1.5, 2.3	49	0.2	−0.5, 2.6
Movements	49	0.07	−4, 6.6	48	0.05	−5.3, 7.6	49	**0.36**	−1.58, 11.7 **
Tone	49	0.22	−0.2, 1.7	48	0.09	−0.8, 1.6	49	**0.27**	−0.05, 1.84 *
Reflexes	49	0.12	−0.9, 2	48	−0.06	−1.47, 2.3	49	**0.25**	−0.2, 2.8 *
Total score	49	0.16	−0.2, 0.7	48	−0.09	−0.38, 0.7	49	**0.2**	0.05, 0.9 **

* *p* < 0.1, ** *p* < 0.05. Statistical significance is presented in bold.
